# *Berkeleyomyces rouxiae* is a causal agent of root rot complex on faba bean (*Vicia faba L*.)

**DOI:** 10.3389/fpls.2022.989517

**Published:** 2022-12-08

**Authors:** Juechen Long, Wenqi Wu, Suli Sun, Yang Shao, Canxing Duan, Yanping Guo, Zhendong Zhu

**Affiliations:** ^1^ Institute of Crop Sciences, Chinese Academy of Agricultural Sciences, Beijing, China; ^2^ Institute of Specialty Crop, Chongqing Academy of Agricultural Sciences, Chongqing, China; ^3^ Linxia Institute of Agricultural Sciences, Linxia, Gansu, China

**Keywords:** *Vicia faba* L., black root rot, *Thielaviopsis basicola*, host range, phylogenetic analysis

## Abstract

Faba bean (*Vicia faba* L.) is an important food and feed legume crop in the world. The root rot complex caused by various pathogens is a main constraint in faba bean production. In April 2021, a severe disease of faba bean with symptoms of black necrosis on roots occurred in experimental fields at the Linxia Institute of Agricultural Sciences, Gansu Province, China. This study aimed to identify the pathogen and evaluate the resistance of faba bean cultivars. The pathogen was isolated from infected soils, and five representative isolates were identified as *Berkeleyomyces rouxiae* based on morphological characteristics, pathogenicity, and molecular phylogenetic analyses. A host range test showed that chickpea, common bean, cowpea, mung bean, rice bean, lentil, and hyacinth bean were susceptible hosts of the faba bean isolate, whereas adzuki bean, pea, and soybean were non-susceptible hosts, and maize and wheat were non-hosts. Identification of resistance among 36 faba bean cultivars was carried out, and six cultivars were found to be moderately resistant to *B. rouxiae*. In this study, we first reported black root rot on faba bean caused by *B. rouxiae*, confirmed and expanded the host range of *B. rouxiae*, and identified resistant faba bean cultivars.

## Introduction

Faba bean (*Vicia faba* L.) is one of the earliest legumes to have been domesticated and ranks fourth in terms of cultivation area among the cool-season food legumes after pea, chickpea, and lentil in this world (Kaur et al., 2014). Faba bean seeds have a high protein content, are a good source of mineral nutrients, and also contain some bioactive compounds (Etemadi et al., 2019). The fresh and dry seeds of the faba bean are used for human consumption, and the dry seeds and straw are also used in livestock feed (Karkanis et al., 2018). Faba bean contributes to the sustainability of cropping systems by fixing atmospheric nitrogen to improve soil fertility, which is often incorporated into various multi-crop and intercropping systems (Jensen et al., 2010; Li et al., 2017). China is a leading faba bean producer with an average planting area of 1.1 million ha and also is one of the largest consumers of faba bean (Ji et al., 2022). In China, the faba bean is mainly grown in the southwest region, Yangtze River basin, and northwest region (Li et al., 2017).

The productivity and quality of faba beans are often significantly reduced by biotic and abiotic stresses. Abiotic factors include frost, heat, waterlogging, and soil salinity and acidity, while biotic factors include diseases, insect pests, and weeds (Stoddard et al., 2010; Maalouf et al., 2018). More than 100 diseases of faba bean have been documented in the world (Yu, 1979; Kumari and Makkouk, 2007), and the number continues to increase (Afshari and Hemmati, 2017; Al-Shahwan et al., 2017; You et al., 2021). Among the diseases of faba bean, the root rot complex has been widely reported worldwide (Sillero et al., 2010). Many fungal and oomycete pathogens have been reported to cause root rot on faba beans, and major pathogens include *Aphanomyces euteiches*, *Fusarium* spp., *Macrophomina phaseolina*, *Pythium* spp., *Rhizoctonia solani* (Kraft et al., 1988; Rubiales and Khazaei, 2022).

*Thielaviopsis basicola* is a cosmopolitan soilborne plant pathogen that attacks more than 230 plant species and causes the disease known as black root rot, and the number of new hosts is increasing (Pereg, 2013; Nakane et al., 2019; Le et al., 2022; Rahnama et al., 2022). The disease is characterized by black necrosis on various parts of the host roots, which leads to stunting, reduced vigor, wilt, and yield loss (Noshad et al., 2006; Coumans et al., 2011). *T. basicola* has a complex taxonomic history and has been assigned different species names. Recently, Nel et al. (2018) performed a phylogenetic analysis using DNA sequence data of six different gene regions and showed that the isolates of *T. basicola* represent two distinct fungal species in the newly described genus *Berkeleyomyces*, *B. basicola* and *B. rouxiae*, which are morphologically indistinguishable but can be distinguished by molecular characterization (Crous et al., 2021; Cavalcante et al., 2022).

Severe root rot on faba bean has occurred in faba bean experimental fields for many years at the Linxia Institute of Agricultural Sciences, Gansu Province, China (35.62 N 103.199 E), which caused stunting, yellowing, premature defoliation, and plant death. However, the pathogens inciting root rot were unclear. In June 2021, several faba bean roots with a symptom of black necrotic lesions were collected from a faba bean field at this Institute. We examined some diseased root epidermal tissues under a microscope and found dark-colored and muriform chlamydospores similar to those of *T. basicola*. The objective of the current study was to confirm the identity of the pathogen causing black root rot on the faba bean using morphological characterization, pathogenicity test, and molecular phylogenetic analysis. In addition, we evaluated the resistance of faba bean cultivars by artificial inoculation.

## Materials and methods

### Isolation of pathogen

In June 2021, soil samples were collected from three experimental plots for faba bean breeding where severe root rot had occurred, and this field has been used for faba bean breeding for 3 years and in rotation with wheat. Three bulk soil samples (2 kg of soil at 5–20 cm depth) were taken from the root zone of plants with root rot. A greenhouse bioassay was used to bait the root rot pathogens from soils. The faba bean cultivar Qinghai 13 which was highly susceptible to root rot in the field was selected for the bioassay. The soil samples were passed through a 10-mesh sieve and filled into 500 mL paper cups with holes at the bottom to about 3/4 of the cup height. For each soil sample, three replicate cups were prepared. Each cup was sown with 5 seeds and watered to saturation. The seeds were also sown in rough vermiculite as controls. The planted cups were kept on a rack in a glasshouse at 22-25°C with natural sunlight and watered as needed. Four weeks after sowing, plants were removed and roots were carefully washed free of soil. Epidermal tissues of black necrotic roots were examined under a microscope, and the diseased root tissues with chlamydospores typical for *T*. *basicola* were used for pathogen isolation using carrot discs (Nel et al., 2019). The diseased lateral roots were excised into 0.5 cm segments and placed on fresh carrot discs in Petri dishes with three layers of moistened filter paper, which were sealed with parafilm and incubated at 25°C. After 7 days, endoconidia were picked from diseased carrot discs with a sterile scalpel for confirmation using a microscope and then diluted to 50 spores/ml in sterile water. Then, 100 μl spore suspension was evenly spread on the 90 mm potato-dextrose agar (PDA; AoBoXing, Biotech, Beijing, China) plates with 25 ug/ml chloramphenicol. The plates were incubated at 25°C for two days, and single colonies were individually transferred to the new PDA plates. Pure single-spore isolates were stored at -80°C on PDA for future use.

### Morphological identification

Five representative isolates (LXBR1 to LXBR5) were selected to determine their identity. The isolates were grown on PDA plates to assess colony characteristics at 25°C. Seven days after incubation, endoconidia, chlamydospores, and phialides were observed and measured under a microscope (Olympus CX 31). Fifty arbitrarily selected structures were measured.

### Pathogenicity and host range tests

The pathogenicity of five representative isolates was tested on the faba bean cultivar Qinghai 13. The inoculum of each isolate was prepared by placing several mycelial plugs (5 mm in diameter) into 100 mL potato dextrose broth, which was incubated for 4 days in an incubation shaker (100 rpm) at 25°C in darkness. The endoconidia suspension was then filtered and adjusted to a final concentration of 1.0 × 10^7^ spores/mL to inoculate faba bean seedlings.

Five seeds were planted in each paper cup (500 mL) filled with fresh vermiculite. The planted cups were placed in the greenhouse for 3 weeks at 22–25°C. The seedlings were uprooted and the roots were washed thoroughly under running tap water, then the roots were soaked in the conidia suspension for 10 minutes, and finally, the seedlings were transplanted into a new cup. There were three replications per isolate, each replicate consisted of three cups, each cup containing three plants, arranged in a completely randomized design (CRD). Plants soaked in sterile water served as the control. The inoculated plants and controls were maintained in a greenhouse at 22–25°C with natural sunlight. The symptoms were investigated 2 weeks after inoculation. The chlamydospores were observed in inoculated roots under the microscope. The pathogen was re-isolated, and the morphology and molecular characteristics of pathogens were identified. The test was repeated twice.

The crops in the host range test included ten legume and two grass crops, namely common bean (*Phaseolus vulgaris* cvs. Biyun 6, Longyundou 29), pea (*Pisum sativum* cvs. Longwan7, Zhongqin 1), chickpea (*Cicer arietinum* cvs. Xinying 1, Xinying 2), cowpea (*Vigna unguiculata* cvs. Liaojiang1, Guijiang 18-11), mung bean (*Vigna radiata* cvs. Elv 1, Zhenglv 8), adzuki bean (*Vigna angularis*; cvs. Jinxiaodou 5, Yuhong 2), rice bean (*Vigna umbellata* cvs. Fandou 1, Hongfandou), lentil (*Lens culinaris* cvs. Yingguozhonglv, Faguo), hyacinth bean (*Lablab purpureus*; cvs. Jiaoda yanhong, Biandou 1), soybean (*Glycine max* cvs. Williams, Zhonghuang13), wheat (*Triticum aestivum* cvs. 197, 198), and maize (*Zea mays* cvs. Zhongdan 1168, Dongdan 6688). LXBR1 was used in this experiment, and the inoculation procedure was the same as in the pathogenicity test. The host range test was arranged in a randomized complete block design (RCBD) with 3 replicate blocks and 3 plants were inoculated in each replicate. The pathogenicity of the isolate was evaluated by investigating the crop symptoms and chlamydospore production on the roots 2 weeks post-inoculation. Typical symptoms and chlamydospores were observed in all three replicates, denoted by “+”, otherwise by “-”. The test was performed twice.

### Molecular phylogenetic analyses

Genomic DNA from the five isolates was extracted from mycelium using the Fungi Genomic DNA Extraction Kit (Solarbio, Beijing, China) following the manufacturer’s instructions. The partial sequence of the internal transcribed spacers (ITS), the ribosomal large subunit (LSU), the minichromosome maintenance complex component 7 (*MCM7*), and the 60S ribosomal protein RPL10 (60S) genes were amplified with primer pairs ITS1/ITS4 (White et al., 1990), LR0R/LR5 (Vilgalys and Hester, 1990), MCM7-for/MCM7-rev de Beer et al., 2014), and 60S-506F/60S-908R (Stielow et al., 2015), respectively. PCR was performed in a final volume of 25 μl containing 9.5 μl water, 1 μl DNA template, 1 μl each primer, and 12.5 μl GoTaq Master Mix PCR (Promega). The amplification cycles were performed by initial denaturation at 94°C for 5 min, followed by 35 cycles of denaturation at 94°C for 30 s, annealing at 56°C (ITS), 53°C (LSU), 58°C (*MCM7*) or 54°C (60S) for 45 s and extension at 72°C for 2 min; and a final extension at 72°C for 7 min. The amplified PCR products were purified and sent to Sangon Biotech (Shanghai) Co., Ltd. for sequencing, using the aforementioned primers.

The resulting sequences were blasted in the National Center for Biotechnology Information (NCBI) Database (http://www.ncbi.nlm.nih.gov) after splicing. The sequences of *B*. *rouxiae* strains and some related species (Nakane et al., 2019; Le et al., 2022) were obtained from the GenBank. The phylogenetic trees for tandem sequences of ITS, LSU, *MCM7*, 60S and a phylogenetic tree for *MCM7* were constructed using the Maximum likelihood method and the Tamura-Nei distance model in MEGA 11 with 1000 bootstrap repeats (Kumar et al., 2016).

### Evaluation of faba bean cultivars for resistance

Thirty-six faba bean cultivars were evaluated for resistance by inoculating with isolate LXBR1. The inoculation procedure was the same as for the pathogenicity test. The experiment was arranged in a RCBD with three replicate blocks, one plastic tray was considered as a block. and each cultivar was randomly assigned to a cup within each plastic tray. The disease severity of each cultivar was investigated 2 weeks after inoculation, and evaluated with a 0 to 5 scale using the criterion of Bodker et al. (1993) as follows: 0 = no symptoms; 1 = 1-10% root or epicotyl area with disease symptoms; 2 = 11-30%; 3 = 31-60%; 4 = 61-90%; and 5 = 91-100%. The disease index (DI) was used to evaluate the resistance of each cultivar. DI was calculated using the following formula: (DI) = (∑ (*n* × *s*)/(*N* × 5)) × 100, where *s* is the score of the disease severity, *n* is the number of plants at that score, and *N* is the total number of plants tested. The resistance was classified based on DI: highly resistant (HR, 0 < DI ≤ 15), resistant (R, 15 < DI ≤ 35), moderately resistant (MR, 35 < DI ≤ 55), susceptible (S, 55 < DI ≤ 75), and highly susceptible (HS, 75 < DI).

The repeated trials were combined for analysis after homogeneity test for variance, and blocks nested within trials were considered as a random component in the mixed model. Square root transformation was carried out for data normalization. Data were used for analyzed using the General Linear Model procedure in SPSS 22.0, Fisher’s least significant difference (LSD) was used for the means comparison. The Anaylsis of Variance (ANOVA) was used to determine any difference in resistance to *B. rouxiae* between cultivars.

## Result

### Isolation and morphological identification

**Figure 1:**
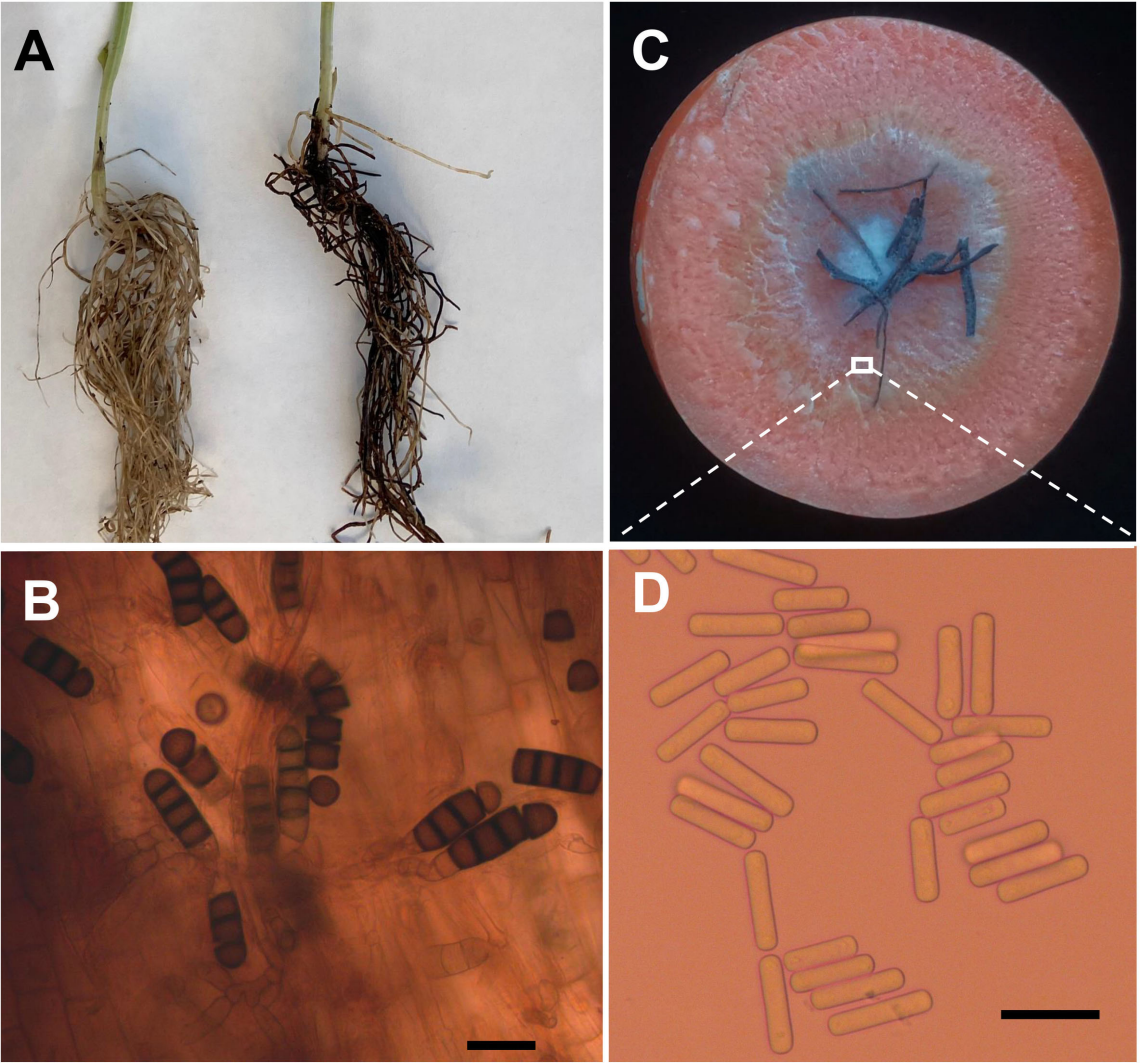
Bioassay of infected soils and isolation of pathogen causing black root on faba bean. **(A)** Seedlings of faba bean from control (left) and infected soils (right). **(B)** Chlamydospores of *Berkeleyomyces rouxiae* produced in faba bean diseased root. **(C)** Carrot slice infected by *Berkeleyomyces rouxiae* on faba bean diseased root. **(D)** Endoconidia of *B rouxiae* produced on carrot slice. (bar = 20μm).

**Figure 2:**
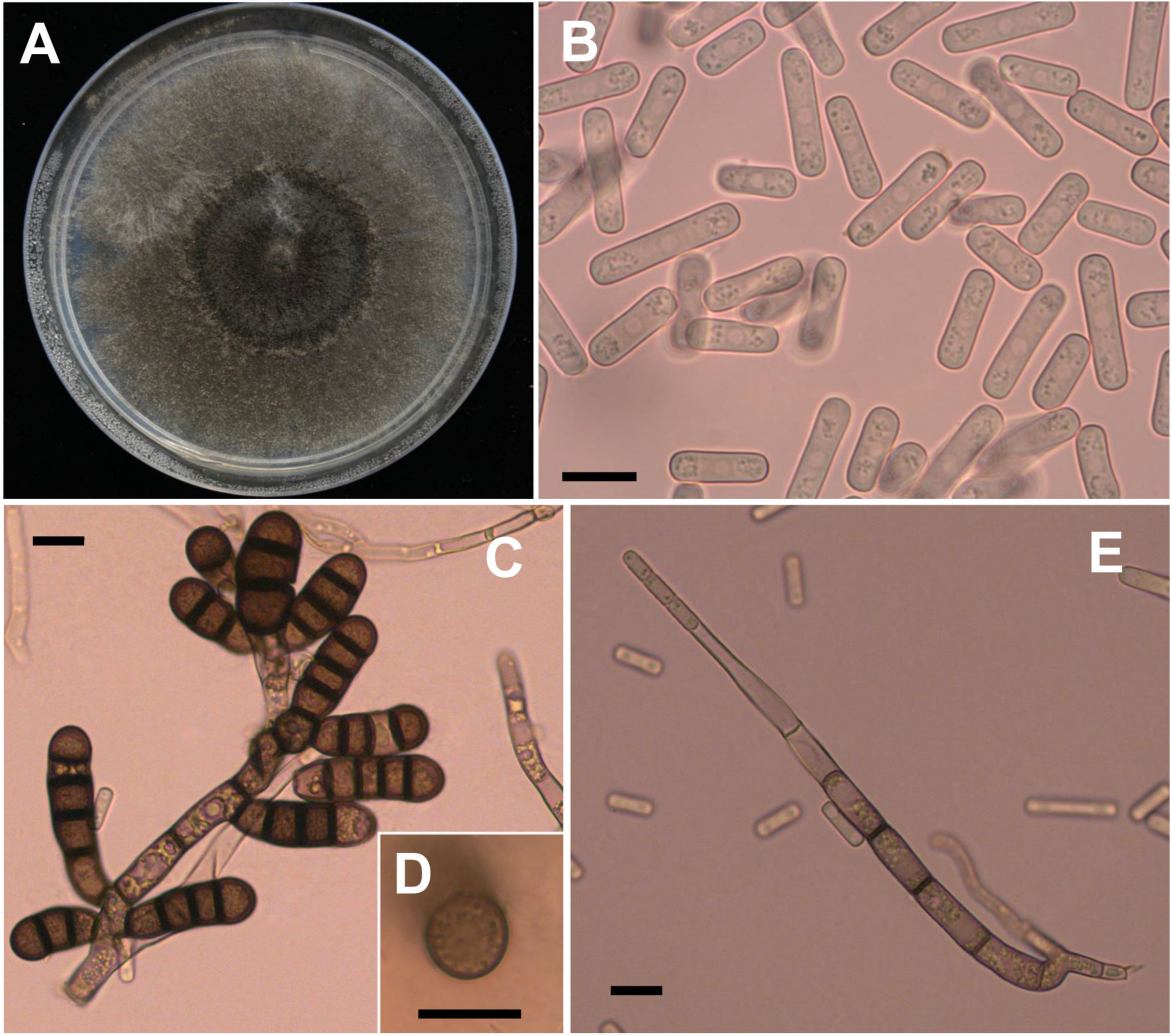
Morphological characteristics of *Berkeleyomyces rouxiae*. **(A)** Colony of *B rouxiae* isolates on PDA after 20 days at 25 °C. **(B)** Endoconidia of *B rouxiae* produced on PDA. **(C, D)** Chlamydospores of *B rouxiae* produced on PDA. **(E)** Phialide of *B rouxiae*. (Bar = 10μm).

Four weeks after faba bean seeds were sown in soil samples, the faba bean seedlings were dwarfed compared to the controls, and severe black necrosis occurred on the roots, while the controls remained healthy (**Figure 1A**). Abundant chlamydospores typical of *T*. *basicola* were observed in the epidermal tissues under the microscope (**Figure 1B**). Seven days after inoculating disease root segments on the carrot discs, many gray hyphae were growing on the discs (**Figure 1C**), and abundant endoconidia were observed under the microscope (**Figure 1D**). Single-spore isolates were obtained by the spore-dilution technique. The colonies of isolates on PDA were initially white, and later the center turned olive green to black following the development of chlamydospores (**Figure 2A**). The average growth rate of isolates on PDA at 25°C was 4 mm/day. Two types of asexual spores, chlamydospores and endoconidia, were produced by the isolates. The chlamydospores were produced in chains containing 3-5 cells (**Figures 2C, D** **)**, dark brown, and 5.6-10.2 μm × 8.6-12.9 μm in size. The endoconidia were hyaline, cylindrical in shape, and produced from phialides, which measured 10.49-25.10 μm × 4.41-6.10 μm in size, and the phialide were measured 5.8-8.2 μm × 112.3-226.7 μm in size (**Figures 2B, E** **)**. The colony characteristics, chlamydospores, and endoconidia size of the isolates were similar to those of *B. rouxiae* (Nakane et al., 2019; Le et al., 2022).

### Pathogenicity and host range tests

Two weeks after inoculation, all five isolates were able to cause stunting of “Qinghai 13” plants and reduced plant vigor, and black necrosis was also observed on the stem base of some plants. Typical black necrotic lesions on plant roots were observed, and chlamydospores were also discovered on the black necrotic lesions under a microscope, while there were no symptoms on the control plants. The results indicated all five isolates were pathogenic to faba bean. The five isolates were also re-isolated from symptomatic lesions to confirm Koch’s postulates. The results of the two experiments were similar.

The results of the host range test revealed that isolate LXBR1 was able to infect all the ten tested legume crops, but was not pathogenic on wheat and maize. Isolate LXBR1 had strong pathogenicity on common bean, chickpea, cowpea, mung bean, rice bean, lentil, and hyacinth bean, where it caused typical black necrosis on roots and produced abundant chlamydospores in the necrotic tissues. However, the isolate did not cause symptoms in soybean, adzuki bean, and pea, and only a few chlamydospores were produced on roots (**Table 1**). The experiment was performed two times, and similar results were obtained.

**Table 1:** Host range test of *Berkeleyomyces rouxiae* isolate from faba bean.

Crop	Cultivar	Infectibility to *B. rouxiae* ^a^	
		black necrosis on root	Sporulationon root
Common bean (*Phaseolus vulgaris*)	Biyun 6	**+**	**+**
Longyundou 29	**+**	**+**
Pea (*Pisum sativum*)	Longwan 7	**-**	**+**
	Zhongqin 1	**-**	**+**
Chickpea (*Cicer arietinum*)	Xinying 1	**+**	**+**
	Xinying 2	**+**	**+**
Cowpea (*Vignaun unguiculata*)	Liaojiang1	**+**	**+**
	Guijiang 18-11	**+**	**+**
Mung bean (*Vigna radiata*)	Elv 1	**+**	**+**
	Zhenglv 8	**+**	**+**
Adzuki bean (*Vigna angularis*)	Jinxiaodou 5	**-**	**+**
	Yuhong 2	**-**	**+**
Rice bean (*Vigna umbellata*)	Fandou 1	**+**	**+**
	Hongfandou	**+**	**+**
Lentil (*Lens culinaris*)	Yingguozhonglv	**+**	**+**
	Faguo	**+**	**+**
Hyacinth bean (*Lablab purpureus*)	Jiaodayanhong	**+**	**+**
	Biandou 1	**+**	**+**
Soybean (*Glycine max*)	Williams	**-**	**+**
	Zhonghuang13	**-**	**+**
Wheat (*Triticum aestivum*)	197	**-**	**-**
	198	**-**	**-**
Maize (*Zea mays* L.)	Zhongdan 1168	**-**	**-**
	Dongdan 6688	**-**	**-**

a, “+”: positive results, “-”: negative results

### Sequence alignment and phylogenetic analyses

**Figure 3:**
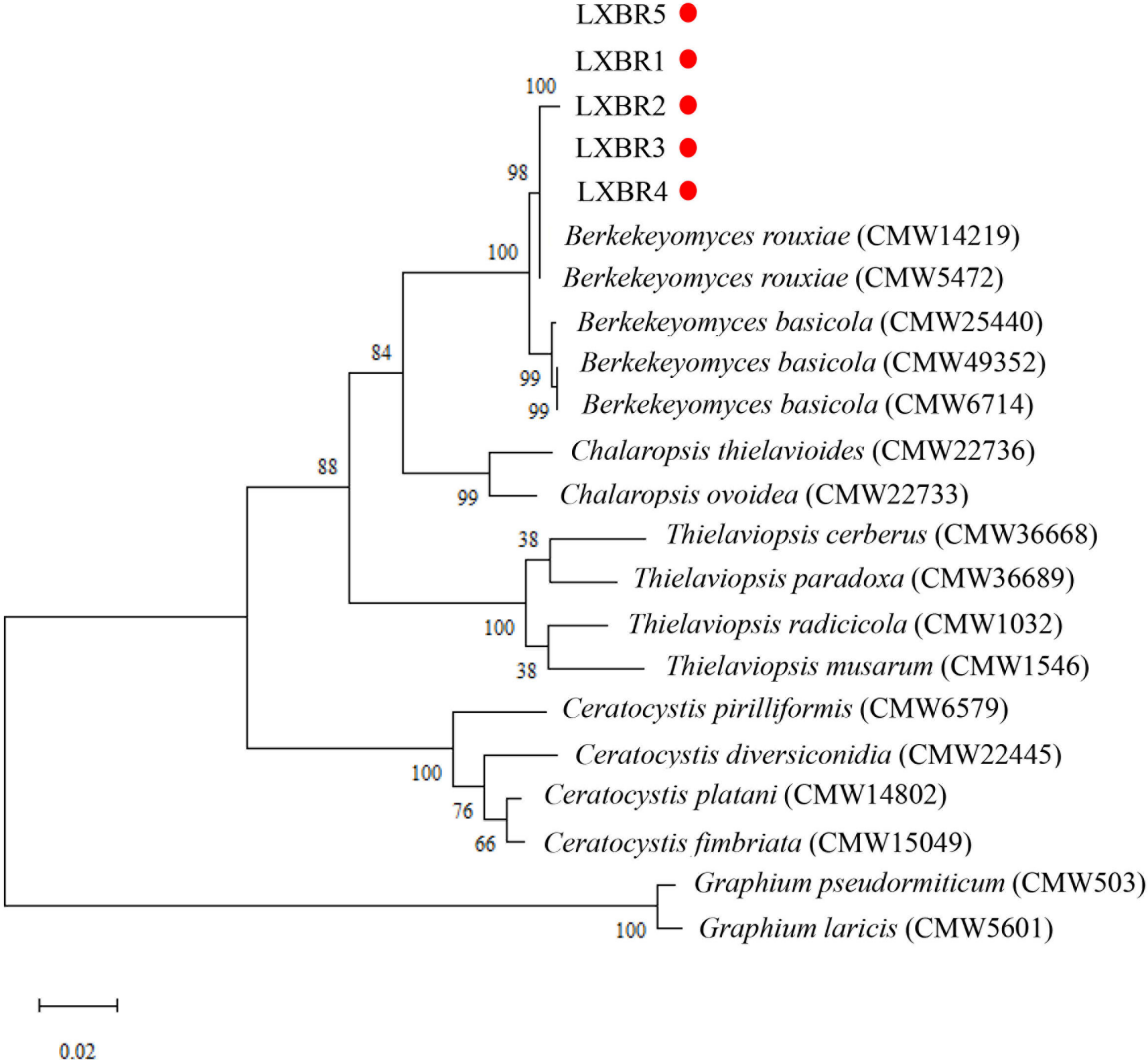
Combined phylogenetic tree based on the ITS, LSU, 60S, and *MCM7* sequences of the *Berkeleyomyces rouxiae* isolates by Maximum-Likelihood method in the MEGA11with bootstrap values estimated by 1000 replicates. Bootstrap support values are indicated in the nodes.

**Figure 4:**
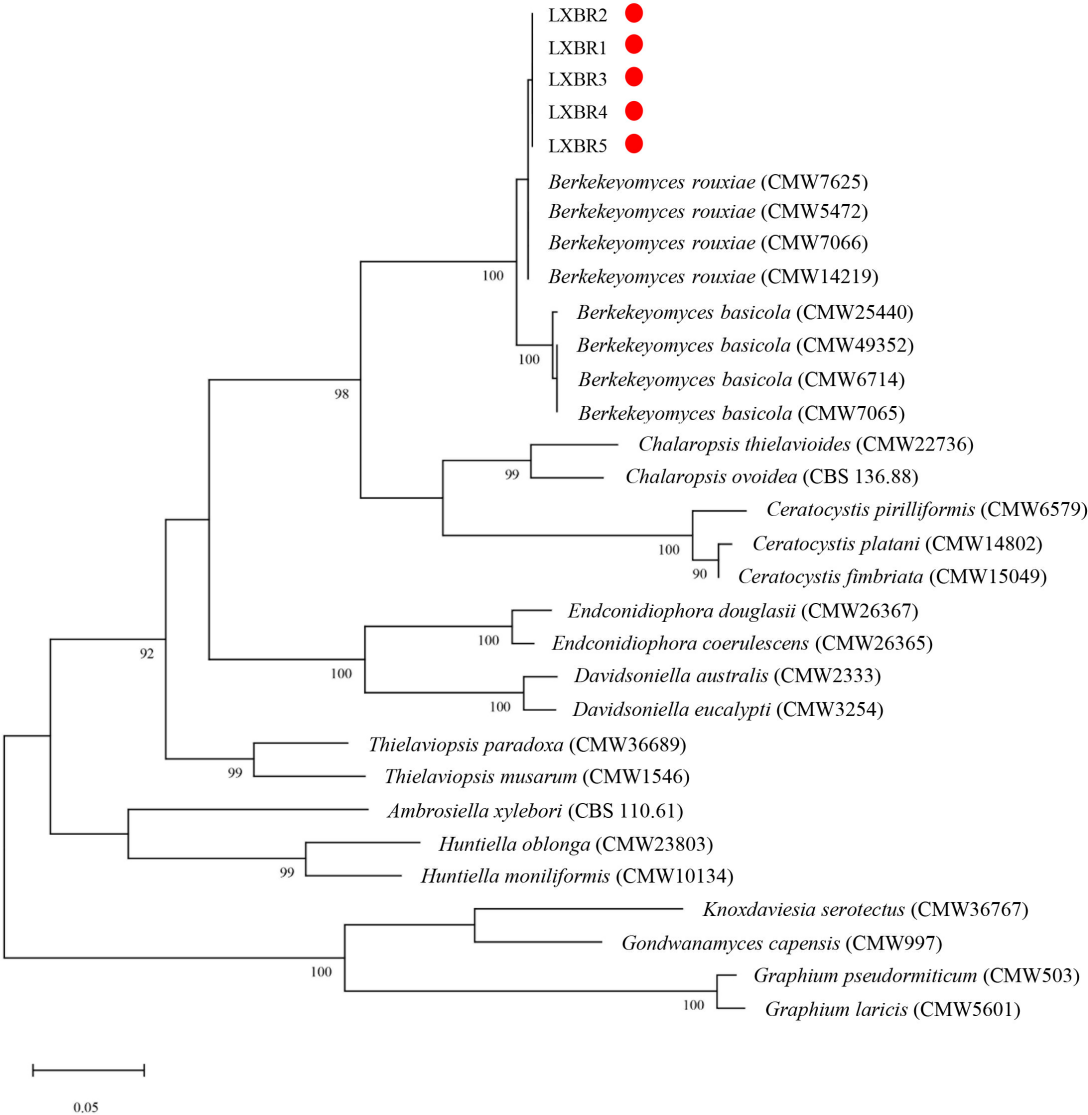
Phylogenetic tree based on the *MCM7* sequences of the *Berkeleyomyces rouxiae* isolates by Maximum-Likelihood method in the MEGA11 with bootstrap values estimated by 1000 replicates. Bootstrap support values are indicated in the nodes.

Partial sequences of the ITS region, 60S, LSU, and *MCM7* genes from the five isolates were sequenced and submitted to NCBI to obtain GenBank accessions (ON679637- ON679641 for ITS; ON679600- ON679604 for LSU; ON711031- ON711035 for 60S; ON711036-ON711040 for *MCM7*). The BLASTn analysis of these sequences showed that the five isolates had high similarity (99 to 100%) with other *B. rouxiae* isolates including the type isolate CMW7625. The maximum likelihood (ML) trees based on tandem sequences of ITS, LSU, *MCM7* and 60S, and *MCM7* of the five isolates from faba bean and related fungal species were constructed respectively (**Figures 3**, **4**), and the five isolates were identified as *B. rouxiae* based on their phylogenetic position.

### Evaluation of faba bean cultivars for resistance

Thirty-six cultivars were identified for resistance to root rot by inoculating with LXBR1 at the seedling stage. Significant differences in resistance were found among the cultivars (P<0.01) (**Table 2**). Resistance levels in these cultivars showed a very broad range of DI ranging from 33.3 to 88.9. Cultivar T18501 with a DI of 33.3 was classified as resistant, and five cultivars T20604, T20605, Edou3203, Edou1103, and Jingdou701 were moderately resistant reactions. The remaining cultivars were susceptible or highly susceptible (**Table 3**).

**Table 2:** Anaylsis of Variance (ANOVA) used to evaluate the significance differences in 36 cultivars resistance to the *Berkeleyomyces rouxiae*.

Variance	SS	df	MS	F	P-value
cultivars	20751.44	35	592.72	13.59	0.0000
Blocks	187.14	2	93.57	2.22	0.116
Error	2949.51	70	42.13		
Total	23888.09	107			

**Table 3:** Resistance identification of thirty-six faba bean cultivars to *Berkeleyomyces rouxiae*.

Cultivar	Source	Disease Index	Resistance
TC3	Jiangsu	82.2	HS
15-147	Jiangsu	68.9	S
Tongqing1	Jiangsu	80.0	HS
T20601	Jiangsu	73.3	S
T20602	Jiangsu	77.8	HS
T20603	Jiangsu	66.7	S
T18501	Jiangsu	33.3	R
T20604	Jiangsu	51.1	MR
T20606	Jiangsu	62.2	S
T20605	Jiangsu	42.2	MR
T09-110-1	Jiangsu	75.6	HS
T16028	Jiangsu	73.3	S
Sucan6	Jiangsu	73.3	S
Chenghu201010-1-1	Sichuan	88.9	HS
Cehnghu25	Sichuan	88.9	HS
Yucan3	Chongqing	84.4	HS
Yucan4	Chongqing	84.4	HS
Zhongcan202	Beijing	73.3	S
Zhongcan201	Beijing	80.0	HS
Haiqing1	Qinghai	82.2	HS
Wancan1	Anhui	66.7	S
1103	Hubei	73.3	S
TC15	Hubei	64.4	S
Edou3203	Hubei	37.8	MR
Edou1103	Hubei	46.7	MR
Yundou1299	Yunnan	80.0	HS
Yundou2883	Yunnan	86.7	HS
Yundou147	Yunnan	73.3	S
Fengdou35	Yunnan	57.8	S
Fengdou36	Yunnan	73.3	S
Jingdou622	Yunnan	57.8	S
Jingdou651	Yunnan	71.1	S
Jingdou650	Yunnan	71.1	S
Jingdou701	Yunnan	53.3	MR
Jingdou614	Yunnan	66.7	S
Jingdou215	Yunnan	68.9	S

## Discussion

Recently, the taxonomic status of the fungus *T*. *basicola* was revised based on molecular phylogenetic analysis, and the isolates from different hosts were classified into two species under a newly described genus *Berkeleyomyces*, namely *B*. *basicola* and *B*. *rouxiae* (Nel et al., 2018). In this study, we identified the agent causing black root rot of faba beans in experimental field plots in Gansu Province, China. Combining morphological, pathogenic, and molecular characteristics, the faba bean isolates were identified as *B. rouxiae* (Nakane et al., 2019; Le et al., 2022). Based on molecular phylogenetic analysis, Nel et al. (2018) classified the pathogens causing black root rot from *Ipomoea batatas* and *P. sativum* as *B*. *rouxiae* (Nel et al., 2018). Our results confirmed *B*. *rouxiae* as a pathogen causing black root rot on the faba bean.

Black root rot caused by *T*. *basicola* has been reported on more than 230 plant species (Pereg, 2013). Reclassification based on molecular phylogenetic analysis has also classified *T*. *basicola* (= *Thielavia basicola*, *Trichocladium basicola*) isolates from *Arachis hypogaea*, *Chamaecytisus* cult. Aura, *Cichorium intybus*, *Citrus* sp., *Daucus carota*, *Euphorbia pulcherrima*, *Eucalyptus regnans*, *E*. *globulus*, *E*. *globulus*, *E*. *nitens*, *Lathyrus odoratus*, *Nicotiana tabacum*, and *Phaseolus vulgaris* as *B*. *rouxiae* (Nel et al., 2018). New hosts of *B. rouxiae* including *Cannabis sativa* (Rahnama et al., 2022), *Cucumis melo* (Wang et al., 2019), *Gossypium hirsutum* (Le et al., 2022), and *Lactuca sativa* (Nakane et al., 2019) were found or confirmed since 2018. In this study, our results showed that *B. rouxiae* isolate LXBR1 from faba bean was pathogenic to chickpea, common bean, cowpea, hyacinth bean, lentil, mung bean, and rice bean, and infective to adzuki bean, pea, and soybean, whereas it could not infect wheat and maize. Previous studies revealed infection of chickpea, common bean, cowpea, faba bean, hyacinth bean, lentil, pea, soybean, and wheat with *T*. *basicola* (= *Thielavia basicola*, *Trichocladium basicola*) (Johnson, 1916; Gayed 1972; Bowden et al., 1985; Monfort et al., 2010; Pereg, 2011), while maize was not infected by *T*. *basicola* (= *Thielavia basicola*) after natural or artificial inoculations (Johnson, 1916; Gayed 1972). Pereg (2011) found that *T*. *basicola* exhibits three modes of interaction with plants: infects roots and causes the disease; infects roots but does not cause disease; and does not infect roots. Based on these three modes, plants could be divided into susceptible hosts, non-susceptible hosts, and non-hosts of *T*. *basicola*. Non-susceptible hosts are those in which chlamydospores of *T*. *basicola* were detected on healthy-looking roots of the plants. In this study, we found that inoculated adzuki bean, pea, and soybean did not develop symptoms on the roots, but chlamydospores were present, suggesting these crops are non-susceptible hosts of *B. rouxiae* from faba bean. Our results confirmed that the 11 legume crops tested were hosts of *B. rouxiae*, and the infection of adzuki bean, mung bean, and rice bean by *B. rouxiae* was recorded for the first time.

*T. basicola* from common bean (= *Thielavia basicola*) and pea (= *Trichocladium basicola*) was renamed as *B. rouxiae* by Nel et al. (2018), our results showed that the common bean was a host of *B. rouxiae*, but pea was a non-susceptible host of *B. rouxiae*. Previous studies had shown differences in the host range of *T. basicola* isolates from different hosts, suggesting that *T. basicola* may exhibit host specificity and preference for different species (Pereg, 2011; Nel et al., 2018). For example, O’Brien and Davis (1994) found that two *T. basicola* (= *Chalara elegan*s) isolates from lettuce were not pathogenic to cotton. Recently *T. basicola* isolates from cotton and lettuce had been re-identified as *B. rouxiae* (Nakane et al., 2019; Le et al., 2022). These results suggest that *B. rouxiae* could have host specificity and preference, but this should be confirmed by host range tests of *B. rouxiae* from different host plants in the future.

Previous studies revealed that resistance levels to *T*. *basicola* were different in several crops such as chickpea (Bhatti and Kraft, 1992), cotton (Wheeler et al., 2000), soybean (Maduewesi and Lockwood, 1976), and tobacco (Miki and Katsuya, 1998). In this study, we evaluated the resistance of 36 faba bean cultivars to *B. rouxiae* by artificial inoculation at the seedling stage. Although none were completely resistant to *B. rouxiae*, one cultivar showed resistance and five were moderately resistant. The cultivation of these resistant cultivars could be an effective means to manage the black root rot of faba bean by contributing to a decrease in disease severity. To our knowledge, this is the first report of *B. rouxiae* causing root rot on faba beans in China and this disease should be paid sufficient attention to due to the serious risk of *B. rouxiae* in faba beans.

## Data availability statement

The datasets presented in this study can be found in online repositories. The names of the repository/repositories and accession number(s) can be found in the article.

## Author contributions

ZZ planned and designed the experiments. JL and WW performed the experiments and wrote the manuscript. YS and YG provided the diseased soil samples for this study. SS, WW, and CD revised the manuscript. All authors contributed to the article and approved the submitted version.

## Funding

This study was supported by the China Agriculture Research System of MOF and MARA (CARS-08), and the Scientific Innovation Program of the Chinese Academy of Agricultural Sciences.

## Conflict of interest

The authors declare that the research was conducted in the absence of any commercial or financial relationships that could be construed as a potential conflict of interest.

## Publisher’s note

All claims expressed in this article are solely those of the authors and do not necessarily represent those of their affiliated organizations, or those of the publisher, the editors and the reviewers. Any product that may be evaluated in this article, or claim that may be made by its manufacturer, is not guaranteed or endorsed by the publisher.
